# Cytokine Gene Polymorphisms in Patients with Chronic Inflammatory Demyelinating Polyneuropathy

**DOI:** 10.3390/cells12162033

**Published:** 2023-08-10

**Authors:** Ivo Bozovic, Vladimir Perovic, Ivana Basta, Stojan Peric, Zorica Stevic, Dusan Popadic, Irena Vukovic, Aleksandar Stojanov, Emina Milosevic

**Affiliations:** 1 Neurology Clinic, University Clinical Center of Serbia, 11000 Belgrade, Serbia; ivo.bozovic20@gmail.com; 2 Institute of Microbiology and Immunology, Faculty of Medicine, University of Belgrade, 11000 Belgrade, Serbia; perovic.vladimir@med.bg.ac.rs (V.P.); dusan.popadic@med.bg.ac.rs (D.P.); irena.vukovic@med.bg.ac.rs (I.V.); 3 Neurology Clinic, University Clinical Center of Serbia, Faculty of Medicine, University of Belgrade, 11000 Belgrade, Serbia; ivanabasta@yahoo.com (I.B.); stojanperic@gmail.com (S.P.); zstevic05@gmail.com (Z.S.); 4 Neurology Clinic, University Clinical Center of Nis, 18000 Nis, Serbia; astojanov1986@gmail.com

**Keywords:** chronic inflammatory demyelinating polyneuropathy, single-nucleotide polymorphisms, cytokines

## Abstract

Innate and adaptive immune responses exert their role in CIDP pathogenesis through cytokine production. Single-nucleotide polymorphisms (SNPs) may alter cytokine gene expression, with a potential influence on the pathogenesis of autoimmune diseases. However, cytokine gene SNPs have not been assessed in CIDP patients yet. We assessed functional SNPs in the genes encoding IL-10 (rs1800896, rs1800871, rs1800872 and rs3024505), IL-6 (rs1800795), TNF (rs1800629 and rs361525), IL-12B (rs3212227), IFN-γ (rs2430561), GM-CSF (rs25882) and IL-17F (rs11465553) in a cohort of 88 CIDP patients and 486 healthy controls (HCs) via qPCR. We found an association of SNP in the *IL10* promotor and CIDP occurrence. Major homozygotes (AA) were more frequent in the HCs compared to CIDP patients (*p* = 0.049), but the GA genotype prevailed among the patients (*p* = 0.032). A lower frequency of the C allele was observed for rs1800871 and rs1800872 in CIDP patients compared to the HCs (*p* = 0.048). A higher proportion of A carriers at position -1082 (rs1800896) (presumed to be a low IL-10 producer) was noted in patients with milder disability (low INCAT). All mild-INCAT patients were C carriers for rs1800871 and rs1800872 in *IL10* (*p* = 0.038). Furthermore, the *IL6* rs1800795 GG genotype was more frequent in patients (*p* = 0.049) and the CG heterozygote in the HCs (*p* = 0.013). Among the CIDP patients, being a G carrier for this SNP was associated with a higher frequency of type 2 diabetes (T2D) compared to being a non-carrier (*p* = 0.032). Our data indicate a possible association of the *IL10* and *IL6* SNPs with CIDP, but also with disease severity and T2D occurrence. Given the paucity of CIDP patients, multicentric studies are necessary to draw definite conclusions on these associations.

## 1. Introduction

Chronic inflammatory demyelinating polyradiculoneuropathy (CIDP) is an acquired immune-mediated neuropathy that is typically characterized by progressive, stepwise, or recurrent symmetric proximal and distal weakness, sensory dysfunction and absent or reduced tendon reflexes in all extremities, developing over at least eight weeks [1]. Although the etiology and precise mechanisms of CIDP pathogenesis are only partially understood, it is considered an autoimmune disease, mediated by both humoral and cellular immunity against self-antigens present in the peripheral nerves [2]. Helper T cell Th1 and Th17 subpopulations have pivotal roles in the pathogenesis of various autoimmune diseases [3]. In CIDP, T cells infiltrate neural connective tissue along with macrophages, releasing cytokines that govern myelin and axonal injury [4,5,6]. Generally, Th1 is a major macrophage activator that functions via IFN-γ secretion, and in turn, it is differentiated by IL-12 produced by macrophages. Macrophages engulf myelin, function as executors of neural destruction and release proinflammatory cytokines such as TNF and IL-6 [7,8]. Moreover, IL-12 in cerebrospinal fluid (CSF) is associated with CIDP occurrence [9]. IL-17 family members and a granulocyte–macrophage colony-stimulating factor (GM-CSF), the Th17 signature cytokines, have a pathological role in the demyelination of the central nervous system (CNS) in multiple sclerosis (MS) [10], and these cells are considered pathological in CIDP as well [11]. On the other hand, T regulatory cells (Treg cells) counterbalance proinflammatory Th1 and Th17 subsets via several mechanisms, such as IL-10 release [12,13]. The number of Treg cells and their suppressor function are impaired in CIDP [14]. Furthermore, autoreactive B cells in CIDP are dependent both on T cell cytokines and direct contact for class switching to pathological IgG production [15]. Yet, the role of IL-10 in CIDP is dual; in addition to its suppressive effect on proinflammatory Th populations, Th2-secreted IL-10 is considered necessary for B cell switching to the IgG4 subclass specific to neurofascin [16,17]. Differential expression in peripheral blood mononuclear cells (PBMC) from CIDP patients and/or cytokine levels found in the CSF and sera further support the significance of a cellular immune response in CIDP pathogenesis [2].

Given the complexity of the immune response in CIDP, it could be that the mechanisms of tissue damage vary patient-to-patient, which may lead to a poor treatment response in a significant portion of cases [16]. Thus, a better understanding of immunopathogenesis is essential to provide guidance towards individually tailored treatment. Despite the notion that genetic background may contribute to overall disease risk [18], disease biology and the response of a particular patient to treatment [19], the genetic background of CIDP is still under-investigated. Single-nucleotide polymorphism (SNP) studies found associations with susceptibility and/or disease traits in several autoimmune disorders [20]. Regarding genetic studies in CIDP, beyond human leukocyte antigen (HLA) region assessment, only a few SNP studies have been conducted [21,22,23,24,25]. In particular, there are no studies exploring the association of CIDP susceptibility and SNP variants affecting the function of cytokines.

Therefore, our aim was to analyze the SNPs of cytokines with previously shown significance in other autoimmune diseases in a cohort of CIDP patients. We focused on SNPs with functional impacts located in cytokines of significance for helper T cell subsets relevant to the pathogenesis of CIDP and genotyped SNPs in genes for: IL-10 (rs1800871, rs1800896, and rs3024505), IL-6 (rs1800795), TNF (rs1800629 and rs361525), IL-12B (rs3212227 and rs6887695), IL-17F (rs11465553) and GM-CSF (rs25882). We identified different genotype distributions between CIDP patients and a healthy population for *IL10* rs1800896, as well as the differences in the allele frequencies of *IL10* rs1800871, and subsequently, rs1800872, an SNP in complete linkage disequilibrium with the latter. However, these discrepancies did not affect haplotype distribution in the *IL10* promotor.

## 2. Materials and Methods

This study included CIDP patients from two Serbian neuromuscular centers: the Neurology Clinic of the University Clinical Center of Serbia and the Neurology Clinic of the Clinical Center of Nis. Patients who regularly visited their neurologists during 2016–2018, or were diagnosed at that time, were sampled. Only patients who fulfilled the EFNS/PNS CIDP diagnostic criteria from 2010 were included in this research [1]. CIDP variants were also established according to the EFNS/PNS criteria. The final number of genetically tested CIDP patients was 88. In addition, 486 blood samples of healthy donors were obtained from the National Blood Transfusion Institute. This study was approved by the Ethical Committee of the Faculty of Medicine, University of Belgrade (No. 17/I-5), in accordance with the Declaration of Helsinki, and informed consent was obtained from all patients and healthy controls before blood sampling.

Sociodemographic and diagnostic data were collected from the patients themselves and their medical records at the time of blood sampling. The disease onset was classified as acute when the peak of the disease was reached within four weeks from onset, subacute if it lasted between 4 and 8 weeks, and chronic if the initial progressive phase lasted more than 2 months [26]. All of these patients, including those with acute and subacute onset, later had additional fluctuations during the disease course, so the diagnosis of chronic immune-mediated neuropathy was clearly confirmed. Preceding factors were considered relevant if they appeared in a range of 3 to up to 42 days before disease onset [27]. The presence of significant comorbid disorders, such as type 2 diabetes mellitus (T2D), different connective tissue disorders and monoclonal gammopathy of undetermined significance (MGUS) (excluding patients with IgM), was noted, as well as therapeutic strategy and treatment response. We used the Medical Research Council sum score (MRC-SS) [28] to evaluate muscle strength. In accordance with the Inflammatory Neuropathy Cause and Treatment (INCAT) disability score [29], the degree of patients’ functionality was established. Disease worsening was considered as an increase of at least 1 point, and improvement as a 1-point decrease, on the adjusted INCAT disability scale and was evaluated at disease onset and at blood sampling time points. INCAT disability was qualified as mild for values of 1–3 and severe for values of 4–6. The CIDP Disease Activity Status (CDAS) scale was used in order to classify CIDP patients in accordance with their disease activity and treatment status. This is a nominal measure which uses broad categories of disease activity, including “cured”, “remission”, “stable active disease”, “improving”, and “unstable active disease” [30].

### 2.1. DNA Extraction and SNP Detection

Genomic DNA was isolated from patients’ peripheral blood using a GeneJET Whole Blood Genomic DNA Purification Mini Kit (Fermentas Thermo Fisher Scientific Inc., Vilnius, Lithuania). Genotyping was performed using a TaqMan genotyping assay (PE, Applied Biosystems Inc., Foster City, CA, USA) with Maxima Probe qPCR Master Mix (Fermentas Thermo Fisher Scientific Inc.) using a RealPlex2 (Eppendorf AG, Hamburg, Germany), under cycling conditions recommended by manufacturer of the oligonucleotide mixture. The cycling conditions were established as recommended by the manufacturer of the oligonucleotide mix or as previously described for *IFNG* rs2430561 [30]. We assessed the functional SNPs of the genes encoding IL-10 (rs1800896, rs1800871 and rs3024505), IL-6 (rs1800795), TNF (rs1800629 and rs361525), IL-12B (rs3212227); IFN-γ (rs2430561), GM-CSF (rs25882) and IL-17F (rs11465553). In addition, the genotypes for *IL10* rs1800872 were inferred from the SNP rs1800871 because these two SNPs are in complete linkage disequilibrium in Caucasians [31,32]. The whole process was explained in detail in our previous publications [31,33,34]. Analysis was performed in 88 CIDP patients and 486 healthy control subjects (HC) for the majority of the analyzed SNPs. Due to technical reasons, for several SNPs of interest, there was a smaller number of HCs.

### 2.2. Statistical Analysis

All results were tested for Hardy–Weinberg equilibrium. Comparisons between genotype and allele frequencies in different populations were assessed using the Chi-square test. *p* values that were less than 0.05 were considered statistically significant. Distributions of allele, genotype and haplotype frequencies between cases and controls were compared via Pearson’s Chi squared test or Fisher’s exact test where appropriate. Two-tailed *p* values, odds ratios (OR) and 95% confidence intervals (CI) were calculated. *p* values less than 0.05 were considered significant. The estimation of haplotype frequencies was carried out via the Expectation-Maximization algorithm using Arlequin 3.5.1.3 [35]. The IBM Statistical Package for Social Sciences, version 20 (IBM SPSS, Chicago, IL, USA), was used for statistical analyses.

## 3. Results

We compared allele and genotype distributions for SNPs with functional impacts within genes of potentially relevant cytokines for CIDP pathogenesis, between HCs and patients with CIDP. Furthermore, we performed a haplotype analysis of genes with more than one genotyped SNP (*IL10* and *TNF*).

The main sociodemographic and clinical features of all the genetically investigated CIDP patients are shown in Table 1. The majority (86.3%) of our 88 patients met the definite diagnostic CIDP EFNS/PNS criteria.

The results of genotyping for all the investigated SNPs in our study population are shown in Table 2. The genotype distributions for the assessed SNPs in the control and patient groups were in Hardy–Weinberg equilibrium, except for the genotype distribution in *IL17F* (rs2430561) both in the patient and control groups, and *IL6* in the patient group.

We noted a statistically significant difference in genotype frequencies between CIDP patients and the HCs for rs1800896 *IL10* polymorphism. The dominant homozygote AA was underrepresented (*p* = 0.049), while the GA genotype had a higher frequency in the patient group (*p* = 0.032). Also, a significantly higher frequency of the T allele was observed for rs1800871, and of the A allele for rs1800872, in *IL10* in patients with CIDP compared to the HCs (*p* = 0.048).

The haplotype distributions were compared for every gene with multiple SNPs assessed, i.e., *IL10* and *TNF*. In *IL10*, three haplotypes with frequencies greater than 1% in patients and controls were identified (Table 3).

The frequencies of *IL10* haplotypes were comparable in the CIDP patients and the healthy controls. The rare genotype (GTA) was omitted from the analysis. We did not find any significant differences in *TNF* dinucleotide haplotype frequencies between patients with CIDP and the HCs.

Another disparity was observed for genotype frequencies of rs1800795 polymorphism in the gene encoding IL-6 (*p* = 0.013 in favor of the GC genotype in HCs and *p* = 0.049 in favor of the GG genotype in CIDP patients). Frequencies of alleles and genotypes of polymorphisms of other investigated genes were not significantly different in CIDP patients compared to the HCs.

We also assessed whether the distributions of allele carriers and genotypes correlated with the clinical characteristics of patients with CIDP. The associations were observed in relation to the presence of T2D in CIDP and to INCAT at nadir, as a measure of disability. G allele carriers for the rs1800795 polymorphism in the gene encoding IL-6 in the group of CIDP patients were more likely to have T2D (96.0% vs. 75.8%, *p* = 0.032, OR = 7.660 (0.954–61.504)), compared to CIDP patients without the G allele. Carriers of the A allele in *IL10* rs1800896 were more frequent in the group of patients with mild disability, as measured via INCAT (91.2% vs. 70.0%, OR = 4.457 (1.335–14.872)). Nevertheless, all the patients with mild INCAT were C carriers for rs1800871 in the *IL10* gene (and consequently, for the C allele in rs1800872, an SNP in complete linkage disequilibrium) compared to 90.0% in the group of patients with severe INCAT (*p* = 0.038, OR = not calculated).

Neither of the investigated cytokine polymorphisms was associated with the mode of CIDP onset (acute, subacute, or chronic), with the treatment response or with CIDP outcome (according to CDAS). Also, we did not find an association of any other assessed characteristic of CIDP with the allele or the genotype of the investigated polymorphism.

## 4. Discussion

The genetic basis of CIDP has not yet been sufficiently investigated, and further studies are still needed to determine the association between a precisely selected group of SNPs (with previously proven significance in several autoimmune disorders), which code for different proteins of the immune system, and CIDP pathogenesis. Although the role of cytokines has already been postulated in the pathogenesis of CIDP [15], no attempts have yet been made to relate the SNPs of genes encoding cytokines and their receptors to CIDP, and different clinical characteristics of the disease. This is the first study in the recent literature that has investigated the association of the alleles and genotypes of *IL10*, *IL6*, *IL12B*, *TNF*, *CSF2*, *IL17F* and *IFNG* genes with CIDP.

IL-10 is considered one of the most important cytokines with anti-inflammatory features; it regulates the functions of different immune components and has an important role in the biology of T and B cells [36]. Polymorphisms located at positions −1082 G/A (rs1800896), −819 C/T (rs800871) and −592 C/A (rs1800872) of the *IL10* promotor are known to be involved in the modulation of IL-10 production [37] and correlated with serum levels of IL-10 [38]. Among these SNPs, rs1800896 has been established as strongly associated with different autoimmune diseases, such as systemic lupus erythematosus, rheumatoid arthritis, and autoimmune thyroid disease [39]. Indeed, a recent meta-analysis of the rs1800896 polymorphism revealed a significant association between autoimmune thyroid disease and the −1082 G allele and the GG + GA genotype of the *IL10* gene [40]. The exact same SNP allele/genotype correlation was observed in other immune-mediated diseases [37]. However, an association between any SNP in the *IL10* gene and CIDP has not yet been analyzed in the current literature. Thus, for the first time, we have shown the association of the *IL10* SNP with CIDP. More precisely, we have observed statistically significant differences in GA and AA genotype frequencies between our CIDP patients and HCs for the rs1800896 polymorphism in the *IL10* gene. Considering another investigated *IL10* gene SNP, rs1800871, and its susceptibility to different autoimmune diseases, Indhumathi et al. observed a notable difference in the frequencies of the minor C allele between psoriasis patients and healthy controls [41]. Similarly, in our study, we found lower C allele frequencies for rs1800871 (and, owing to complete linkage disequilibrium, also for rs1800072) in CIDP patients in comparison to the HCs, indicating that the *IL10* gene SNP might play a role in CIDP susceptibility.

Turner et al. found an association of the above-mentioned SNP in the *IL10* promotor with the amount of secreted cytokine upon the in vitro stimulation of peripheral blood mononuclear cells by concanavalin A [32]. Specifically, A allele carriers at position −1082 (rs1800896) had lower IL-10 production than persons with an absent A allele. Given the putative anti-inflammatory role of IL-10, the finding of a higher proportion of A carriers (presumed to be a low IL-10 producer) in a subgroup of CIDP patients with low INCAT is somewhat unexpected. However, the reports from Luomala et al. [42] and Schotte et al. [43] on the rs1800896 *IL10* SNP in other neurological autoimmune disorders, MS and RA, respectively, are in line with our findings. For instance, Luomala et al. determined a protective role of the AG genotype of the *IL10* gene against severe MS forms, suggesting that this polymorphism might affect disease severity rather than the onset of the disease [42]. On the other hand, Schotte et al. suggested that low IL-10 producers were associated with better immuno-treatment responses in patients with RA, which could at least partially contribute to a milder disease presentation [43].

Nevertheless, the role of IL-10 in CIDP has not been elucidated and further investigations are still needed. Data from animal models on the role of IL-10 in CIDP pathogenesis are conflicting. On one hand, B7-2-deficient NOD mice spontaneously develop autoimmune polyneuropathy associated with a decrease in IL-10 [44,45]. On the other hand, IL-10 production was associated with disease development in several animal models of CIDP, as well as with a CIDP variant with anti-neurofascin 155 antibodies and polyneuropathy associated with IgM monoclonal gammopathy with anti-MAG antibodies [46,47,48,49]. Moreover, IL-10 levels were higher in the active phase than in CIDP remission [50]. Based on the available literature, it is not clear whether higher IL-10 reflects a pathogenic Th2 response, regulatory response or a transient counterbalance to nerve-damaging inflammation. Therefore, the roles of IL-10 and genotype-to-phenotype relations in CIDP warrant further exploration.

Due to its prominent proinflammatory function, IL-6 is regarded as a key player in the regulation of the immune response, and dysregulation of the IL-6/IL-6R system has already been associated with the pathogenesis of several autoimmune diseases [51,52]. An SNP in the promotor region of *IL6* at position −174 (rs1800975) is the functional polymorphism, and the G allele has been associated with higher IL-6 production [53]. We found a lower frequency of heterozygotes in the group of patients with CIDP compared to the HCs and a higher frequency of GG homozygotes for this SNP. The latter was in line with the association of the G allele with increased risk for the development of systemic-onset juvenile chronic arthritis [53]. However, in rheumatoid arthritis studies in both Caucasian and non-Caucasian populations, a significantly higher frequency of the rs1800795 CC genotype was observed among patients with rheumatoid arthritis compared to healthy controls [18,54].

The assessment of this SNP and susceptibility to T2D yielded opposing data, but the C allele was a risk factor for higher BMI and insulin resistance [55,56,57,58]. However, the most current meta-analysis refuted the association of *IL6* rs1800795 and T2D risk [59]. In our study, CIDP carriers of the G allele of rs1800795 polymorphism in the *IL6* gene more frequently had T2D compared to the other CIDP patients. A recent European multicenter study showed a twofold increased relative risk of T2D in CIDP patients when compared with the general population [60]. Although the mechanisms promoting CIDP onset in patients with T2D are uncertain, it seems that chronic hyperglycemia, in susceptible individuals, might promote a prolonged proinflammatory state and facilitate CIDP onset [60].

Besides the above-mentioned association between the G allele carriers for rs1800795 polymorphism in the *IL6* gene and T2D presence in CIDP, we also observed a correlation between INCAT at nadir (a measure of disability) and rs1800896 and rs1800871 polymorphisms in the *IL10* gene. During this analysis, a lower (than initially expected) INCAT score in our cohort was noted at the time of investigation. This finding could be interpreted in two main ways, and the first one was the early establishment of CIDP diagnosis in our patients. As per our recent publication, the mean delay in referring a patient with suspicion of CIDP from secondary to tertiary health centers in Serbia is quite short (lasting, on average, eight months) [61]. The second reason for having a low INCAT score might be the timely and adequate immune therapy of these patients, which typically leads to significant improvement in these patients. However, in order to exclude a confounding variable of treatment regarding the association between SNPs and INCAT, the “treatment response” variable was introduced, which represents a relative INCAT change rather than the absolute INCAT value. Finally, we did not find any other association of SNPs with the clinical characteristics of CIDP, including the type of disease onset, CIDP variants and treatment response. Although these associations would be of immense clinical interest, genotype-to-phenotype translation remained beyond reach, even in extensive multicentric studies in multiple sclerosis, for instance, presumably due to polygenic background and multifactorial pathogenesis [62].

The main limitations of our study are the fact that we could not exclude eventual ethnic specificities of the Serbian population regarding some of the analyzed SNPs, and the relatively small sample size. Despite these limitations, this is still the first study that has evaluated the relevance of cytokine polymorphisms in a representative number of patients diagnosed with this rare disorder. Considering the prevalence of CIDP, future studies ought to be multicentric studies to rule out the contribution of SNPs in cytokine genes in this disease.

## 5. Conclusions

Our results expand the data regarding the role of several investigated functional cytokine polymorphisms in CIDP and identify the *IL10* promotor and *IL6* gene SNPs as possible CIDP susceptibility biomarkers. These SNPs were associated with a few of the clinical characteristics of patients (INCAT at nadir and the presence of T2D). However, further research is necessary to confirm our findings and to identify potential genetic biomarkers of disease activity and CIDP outcome predictors. Eventually, this can improve our knowledge regarding CIDP pathogenesis and lead to new possibilities for the treatment of this chronic and debilitating disease.

## Figures and Tables

**Table 1:** Sociodemographic and clinical features of investigated CIDP patients at time of genetic testing (*n* = 88).

CIDP Features	At Testing
Male gender (***n*** (%))	61 (69.3%)
Age at onset (years ± SD)	53.7 ± 14.2
Disease duration (months ± SD)	78.2 ± 76.0
EFNS/PNS diagnostic criteria (***n*** (%)) Definite CIDP	76 (86.3%)
Definite CIDP	76 (86.3%)
Probable CIDP	6 (6.8%)
Disease onset (***n*** (%)) Unknown	3 (3.4%)
Acute	12 (13.6%)
Subacute	8 (9.1%)
Chronic	65 (73.9%)
Precipitating factor (***n*** (%)) Unknown	2 (2.3%)
None	70 (79.5%)
Respiratory infection	6 (6.8%)
Gastrointestinal infection	4 (4.5%)
Other	6 (6.8%)
INCAT disability scale at disease nadir Upper limbs	1.6 ± 1.0
Lower limbs	1.8 ± 1.3
Total	3.4 ± 2.0
INCAT disability at sampling Upper limbs	0.7 ± 0.9
Lower limbs	1.2 ± 1.3
Total	1.9 ± 1.9
CIDP variants (***n*** (%)) Typical	57 (64.8%)
Pure sensory	8 (9.1%)
Pure motor	8 (9.1%)
Focal	3 (3.4%)
DADS	9 (10.2%)
LSS	1 (1.2%)
Comorbid disorders (***n*** (%)) T2D	25 (28.4%)
MGUS	14 (15.9%)
Connective tissue disease	1 (1.1%)
Therapy (any time during disease course) (***n*** (%)) Oral prednisone	68 (77.3%)
Pulsed methylprednisolone	17 (19.3%)
IVIg	34 (38.6%)
PLEx	7 (8.0%)
Immunosuppressant drugs	17 (19.3%)
Treatment response (***n*** (%)) Further worsening	17 (19.3%)
Partial	22 (25.0%)
Good	44 (50.0%)
Unknown	5 (5.7%)

CIDP—chronic inflammatory demyelinating polyneuropathy, DADS—distal acquired demyelinating symmetric neuropathy, INCAT—Inflammatory Neuropathy Cause and Treatment, IVIg—intravenous immunoglobulin, LSS—Lewis-Sumner syndrome, EFNS/PNS—European Federation of Neurological Societies/Peripheral Nerve Society, T2D—type 2 diabetes mellitus, MGUS—monoclonal gammopathy of undetermined significance, NCS—nerve conduction studies, PLEx—plasma exchange.

**Table 2:** Allele and genotype frequencies of SNPs in controls and patients with CIDP.

Gene (SNP)	HCs (*n* = 486) *n* (%)	CIDP Patients (*n* = 88) *n* (%)	*p* Value	OR (95% CI)
*IL10* (rs1800896) Allele G A Genotype GG GA AA	972 409 (42.1%) 563 (57.9%) 486 91 (18.7%) 227 (46.7%) 168 (34.6%)	176 82 (46.6%) 94 (53.4%) 88 15 (17.0%) 52 (59.1%) 21 (23.9%)	0.265 0.708 0.032 0.049	1.201 (0.870–1.658) 0.892 (0.489–1.626) 1.648 (1.040–2.613) 0.593 (0.351–1.002)
*IL10* (rs1800871) Allele C T Genotype CC CT TT	972 250 (25.7%) 722 (74.3%) 486 34 (7.0%) 182 (37.4%) 270 (55.6%)	176 33 (18.7%) 143 (81.3%) 88 3 (3.4%) 27 (30.7%) 58 (65.9%)	0.048 0.207 0.225 0.071	0.666 (0.444–0.999) 0.469 (0.141–1.562) 0.739 (0.453–1.205) 1.547 (0.961–2.489)
*IL10* (rs1800872) Allele C A Genotype CC CA AA	972 250 (25.7%) 722 (74.3%) 486 34 (7.0%) 182 (37.4%) 270 (55.6%)	176 33 (18.7%) 143 (81.3%) 88 3 (3.4%) 27 (30.7%) 58 (65.9%)	0.048 0.207 0.225 0.071	0.666 (0.444–0.999) 0.469 (0.141–1.562) 0.739 (0.453–1.205) 1.547 (0.961–2.489)
*IL10* (rs3024505) Allele A G Genotype AA AG GG	970 145 (14.9%) 825 (85.1%) 485 10 (2.0%) 125 (25.8%) 350 (72.2%)	176 26 (14.8%) 150 (85.2%) 88 3 (3.4%) 20 (22.7%) 65 (73.9%)	1.000 0.433 ^†^ 0.543 0.740	0.986 (0.627–1.550) 1.676 (0.452–6.218) 0.847 (0.494–1.451) 1.090 (0.651–1.825)
*IL6* (rs1800795) Allele G C Genotype GG CG CC	972 587 (60.4%) 385 (39.6%) 486 173 (35.6%) 241 (49.6%) 72 (14.8%)	176 113 (64.2%) 63 (35.8%) 88 41 (46.6%) 31 (35.2%) 16 (18.2%)	0.340 0.049 0.013 0.420	1.176 (0.842–1.643) 1.578 (0.998–2.495) 0.553 (0.345–0.886) 1.278 (0.704–2.321)
*IL12B* (rs3212227) Allele G T Genotype GG GT TT	456 82 (18.0%) 374 (82.0%) 228 6 (2.6%) 70 (30.7%) 152 (66.7%)	176 40 (22.7%) 136 (77.3%) 88 6 (6.8%) 28 (31.8%) 54 (61.4%)	0.175 0.101 ^†^ 0.841 0.374	1.341 (0.876/2.054) 2.707 (0.849–8.633) 1.053 (0.620–1.789) 0.794 (0.477–1.322)
*TNF* (rs1800629) Allele A G Genotype AA AG GG	972 130 (13.4%) 842 (86.6%) 486 6 (1.2%) 118 (24.3%) 362 (74.5%)	176 26 (14.8%) 150 (85.2%) 88 1 (1.1%) 24 (27.3%) 63 (71.6%)	0.617 1.000 ^†^ 0.548 0.572	1.123 (0.712-1.770) 0.919 (0.109–7.733) 1.169 (0.700–1.953) 0.863 (0.520–1.432)
*TNF* (rs361525) Allele A G Genotype AA AG GG	972 31 (3.2%) 941 (96.8%) 486 0 (0%) 31 (6.4%) 455 (93.6%)	176 7 (4.0%) 169 (96.0%) 88 0 (0%) 7 (8.0%) 81 (92.0%)	0.590 1.000 ^†^ 0.584 0.584	1.257 (0.545–2.902) NA 1.268 (0.540–2.978) 0.788 (0.336–1.851)
*CSF2* (rs25882) Allele T C Genotype TT TC CC	966 763 (79.0%) 203 (21.0%) 483 301 (62.4%) 161 (33.3%) 21 (4.3%)	176 137 (77.8%) 39 (22.2%) 88 54 (61.4%) 29 (32.9%) 5 (5.7%)	0.729 0.862 1.000 ^†^ 0.783 ^†^	0.934 (0.634–1.378) 0.960 (0.602–1.532) 0.983 (0.606–1.593) 1.325 (0.486–3.613)
*IL17F* (rs11465553) Allele T C Genotype TT TC CC	848 37 (4.4%) 811 (95.6%) 424 4 (1.0%) 29 (6.8%) 391 (92.2%)	176 3 (1.7%) 173 (98.3%) 88 0 (0%) 3 (3.4%) 85 (96.6%)	0.098 0.607 ^†^ 0.332 ^†^ 0.173 ^†^	0.380 (0.116–1.247) NA 0.481 (0.143–1.615) 2.391 (0.716–7.979)
*IFNG* (rs2430561) Allele A T Genotype AA AT TT	518 276 (53.3%) 242 (46.7%) 259 74 (28.6%) 128 (49.4%) 57 (22.0%)	176 97 (55.1%) 79 (44.9%) 88 24 (27.3%) 49 (55.7%) 15 (17.0%)	0.671 0.823 0.310 0.322	1.077 (0.764–1.518) 0.937 (0.546–1.610) 1.286 (0.791–2.091) 0.728 (0.388–1.365)

*p* values were calculated using Chi-squared test, except for ^†^, where Fisher’s exact test was used. HC—healthy control subjects, CIDP—patients with chronic inflammatory demyelinating polyneuropathy, NA—not applicable.

**Table 3:** Haplotype frequencies for *IL10* in HCs and patients with CIDP.

*IL10* Haplotypes	HCs ^†^ (*n* = 486) *n* (%)	CIDP Patients (*n* = 88) *n* (%)	*p* Value	OR (95% CI)
**GCC**	406 (41.8)	82 (46.6)	0.233	1.216 (0.881–1.679)
**ACC**	316 (32.5)	61 (34.6)	0.578	1.101 (0.785–1.544)
**ATA**	247 (25.4)	33 (18.7)	0.058	0.677 (0.452–1.016)
Total	972	176		

Haplotype analysis was inferred for rs1800. HC—healthy control subjects, CIDP—patients with CIDP, OD—odds ratio, CI—confidence interval, NS—not significant; *p* values were calculated using Chi-square test. ^†^ *p* values were not calculated for haplotypes with frequencies less than 1% (i.e., GTA genotype, n = 3 in HCs).

## Data Availability

The data that support the findings of this study are available on request from the corresponding author. The data are not publicly available due to privacy and ethical restrictions.
